# Supplementary material for: Studying Chondrichthyans Using Baited Remote Underwater Video Systems: A Review
Source: Animals (Basel). 2024 Jun 25;14(13):1875. doi: 10.3390/ani14131875 (PMC11240523; doi:10.3390/ani14131875)
Supplement: Supplementary file 1 [file animals-14-01875-s001.zip › animals-2965363-supplementary.pdf]

animals

Table S1. Species surveyed with BRUVs. Family, number of papers in which the species was studied and the incidence rate are given.

Copyright: © 2024 by the authors. Licensee MDPI, Basel, Switzerland. This article is an open access article distributed under the terms and conditions of the Creative Commons Attribution (CC BY) license (https://creativecommons.org/licenses/by/4.0/).

Family	Species	n° papers	Incidence rate (%)
Carcharhinidae	*Galeocerdo cuvier*	27	32.5
Carcharhinidae	*Carcharhinus amblyrhynchos*	20	24.1
Carcharhinidae	*Carcharhinus limbatus/tilstoni*	19	22.9
Carcharhinidae	*Carcharhinus melanopterus*	19	22.9
Sphyrnidae	*Sphyrna lewini*	18	21.7
Carcharhinidae	*Triaenodon obesus*	18	21.7
Sphyrnidae	*Sphyrna mokarran*	16	19.3
Carcharhinidae	*Carcharhinus albimarginatus*	14	16.9
Carcharhinidae	*Carcharinus perezi*	12	14.5
Ginglymostomatidae	*Ginglymostoma cirratum*	12	14.5
Carcharhinidae	*Carcharhinus plumbeus*	11	13.3
Ginglymostomatidae	*Nebrius ferrugineus*	10	12.0
Carcharhinidae	*Negaprion acutidens*	10	12.0
Carcharhinidae	*Carcharhinus falciformis*	9	10.8
Carcharhinidae	*Carcharhinus galapagensis*	8	9.6
Lamnidae	*Carcharodon carcharias*	8	9.6
Triakidae	*Galeorhinus galeus*	8	9.6
Carcharhinidae	*Negaprion brevirostris*	8	9.6
Carcharhinidae	*Carcharhinus leucas*	6	7.2
Dasyatidae	*Dasyatis brevicaudata*	7	8.4
Stegostomatidae	*Stegostoma fasciatum*	7	8.4
Dasyatidae	*Taeniura meyeni*	7	8.4
Carcharhinidae	*Carcharhinus brachyurus*	6	7.2
Hexanchidae	*Notorynchus cepedianus*	6	7.2
Dasyatidae	*Taeniura lymma*	6	7.2
Dasyatidae	*Bathytoshia brevicaudata*	5	6.0

Scyliorhinidae	*Haploblepharus edwardsii*	5	6.0
Heterodontidae	*Heterodontus portusjacksoni*	5	6.0
Triakidae	*Mustelus mustelus*	5	6.0
Myliobatidae	*Myliobatis australis*	5	6.0
Scyliorhinidae	*Poroderma africanum*	5	6.0
Rhinidae	*Rhina ancylostoma*	5	6.0
Rhincodontidae	*Rhincodon typus*	5	6.0
Triakidae	*Triakis megalopterus*	5	6.0
Aetobatidae	*Aetobatus narinari*	4	4.8
Carcharhinidae	*Carcharhinus acronotus*	4	4.8
Carcharhinidae	*Carcharhinus amboinensis*	4	4.8
Carcharhinidae	*Carcharhinus sorrah*	4	4.8
Scyliorhinidae	*Haploblepharus pictus*	4	4.8
Dasyatidae	*Himantura uarnak*	4	4.8
Dasyatidae	*Hypanus americanus*	4	4.8
Lamnidae	*Isurus oxyrinchus*	4	4.8
Myliobatidae	*Myliobatis aquila*	4	4.8
Dasyatidae	*Pateobatis fai*	4	4.8
Scyliorhinidae	*Poroderma pantherinum*	4	4.8
Carcharhinidae	*Prionace glauca*	4	4.8
Squalidae	*Squalus acanthias*	4	4.8
Rhinobatidae	*Acroteriobatus annulatus*	3	3.6
Carcharhinidae	*Carcharhinus brevipinna*	3	3.6
Carcharhinidae	*Carcharhinus coatesi*	3	3.6
Hemiscylliidae	*Chiloscyllium punctatum*	3	3.6
Dasyatidae	*Dasyatis thetidis*	3	3.6
Hemigaleidae	*Hemigaleus australiensis*	3	3.6
Hemigaleidae	*Hemipristis elongata*	3	3.6
Carcharhinidae	*Loxodon macrorhinus*	3	3.6
Mobulidae	*Mobula birostris*	3	3.6
Triakidae	*Mustelus antarcticus*	3	3.6
Orectolobidae	*Orectolobus maculatus*	3	3.6
Dasyatidae	*Pastinachus ater*	3	3.6
Carcharhinidae	*Rhizoprionodon acutus*	3	3.6
Carcharhinidae	*Rhizoprionodon taylori*	3	3.6
Squalidae	*Squalus megalops*	3	3.6
Rhinobatidae	*Trygonorrhina fasciata*	3	3.6
Dasyatidae	*Urogymnus asperrimus*	3	3.6
Dasyatidae	*Urogymnus granulatus*	3	3.6

Myliobatidae	*Aetobatus ocellatus*	2	2.4
Dasyatidae	*Bathytoshia lata*	2	2.4
Callorhinchidae	*Callorhinchus capensis*	2	2.4
Carcharhinidae	*Carcharhinus dussumieri*	2	2.4
Carcharhinidae	*Carcharhinus macloti*	2	2.4
Odontaspididae	*Carcharias taurus*	2	2.4
Scyliorhinidae	*Cephaloscyllium isabellum*	2	2.4
Scyliorhinidae	*Cephaloscyllium laticeps*	2	2.4
Dasyatidae	*Dasyatis chrysonota*	2	2.4
Dasyatidae	*Dasyatis pastinaca*	2	2.4
Triakidae	*Furgaleus macki*	2	2.4
Glaucostegidae	*Glaucostegus halavi*	2	2.4
Glaucostegidae	*Glaucostegus typus*	2	2.4
Gymnuridae	*Gymnura natalensis*	2	2.4
Scyliorhinidae	*Halaelurus natalensis*	2	2.4
Hemiscylliidae	*Hemiscyllium ocellatum*	2	2.4
Hexanchidae	*Hexanchus griseus*	2	2.4
Dasyatidae	*Himantura australis*	2	2.4
Dasyatidae	*Himantura leoparda*	2	2.4
Mobulidae	*Mobula japanica*	2	2.4
Triakidae	*Mustelus canis*	2	2.4
Dasyatidae	*Pateobatis jenkinsii*	2	2.4
Dasyatidae	*Pteroplatytrygon violacea*	2	2.4
Rajidae	*Raja straeleni*	2	2.4
Rhinobatidae	*Rhinobatos hynnicephalus*	2	2.4
Rajidae	*Rostroraja alba*	2	2.4
Sphyrnidae	*Sphyrna zygaena*	2	2.4
Squalidae	*Squalus griffini*	2	2.4
Potamotrygonidae	*Styracura schmardae*	2	2.4
Urolophidae	*Trygonoptera testacea*	2	2.4
Urotrygonidae	*Urobatis jamaicensis*	2	2.4
Urotrygonidae	*Urolophus halleri*	2	2.4
Rhinobatidae	*Acroteriobatus leucospilus*	1	1.2
Myliobatidae	*Aetobatus laticeps*	1	1.2
Myliobatidae	*Aetomylaeus vespertilio*	1	1.2
Alopiidae	*Alopias pelagicus*	1	1.2
Rhinobatidae	*Aptychotrema rostrata*	1	1.2
Scyliorhinidae	*Atelomycterus fasciatus*	1	1.2
Scyliorhinidae	*Aulohalaelurus labiosus*	1	1.2
Arhynchobatidae	*Bathyraja shuntovi*	1	1.2

Carcharhinidae	*Carcharhinus amblyrhynchoides*	1	1.2
Carcharhinidae	*Carcharhinus cautus*	1	1.2
Carcharhinidae	*Carcharhinus longimanus*	1	1.2
Carcharhinidae	*Carcharhinus obscurus*	1	1.2
Carcharhinidae	*Carcharhinus sealei*	1	1.2
Centrophoridae	*Centrophorus squamosus*	1	1.2
Etmopteridae	*Centroscyllium fabricii*	1	1.2
Somniosidae	*Centroscymnus coelolepis*	1	1.2
Somniosidae	*Centroscymnus owstoni*	1	1.2
Hemiscylliidae	*Chiloscyllium arabicum*	1	1.2
Squalidae	*Cirrhigaleus australis*	1	1.2
Dalatiidae	*Dalatias licha*	1	1.2
Centrophoridae	*Deania calcea*	1	1.2
Rajidae	*Dentiraja lemprieri*	1	1.2
Narcinidae	*Diplobatis ommata*	1	1.2
Rajidae	*Dipturus innominatus*	1	1.2
Rajidae	*Dipturus whitleyi*	1	1.2
Etmopteridae	*Etmopterus baxteri*	1	1.2
Etmopteridae	*Etmopterus bigelowi*	1	1.2
Etmopteridae	*Etmopterus molleri*	1	1.2
Orectolobidae	*Eucrossorhinus dasypogon*	1	1.2
Ginglymostomatidae	*Ginglymostoma unami*	1	1.2
Pseudotriakidae	*Gollum attenuatus*	1	1.2
Gymnuridae	*Gymnura poecilura*	1	1.2
Hemigaleidae	*Hemigaleus microstoma*	1	1.2
Hemigaleidae	*Hemipristis elongatus*	1	1.2
Hemiscylliidae	*Hemiscyllium trispeculare*	1	1.2
Triakidae	*Hemitriakis abdita*	1	1.2
Triakidae	*Hemitriakis falcata*	1	1.2
Dasyatidae	*Himantura fai*	1	1.2
Chimaeridae	*Hydrolagus affinis*	1	1.2
Dasyatidae	*Hypanus berthalutzae*	1	1.2
Dasyatidae	*Hypanus guttatus*	1	1.2
Dasyatidae	*Hypanus longus*	1	1.2
Dasyatidae	*Hypanus marianae*	1	1.2
Rajidae	*Leucoraja erinacea*	1	1.2
Rajidae	*Leucoraja ocellata*	1	1.2
Dasyatidae	*Megatrygon microps*	1	1.2
Mobulidae	*Mobula alfredi*	1	1.2

Mobulidae	*Mobula kuhlii*	1	1.2
Mobulidae	*Mobula munkiana*	1	1.2
Mobulidae	*Mobula tarapacana*	1	1.2
Triakidae	*Mustelus lenticulatus*	1	1.2
Myliobatidae	*Myliobatis californica*	1	1.2
Myliobatidae	*Myliobatis tenuicaudatus*	1	1.2
Narcinidae	*Narcine bancroftii*	1	1.2
Narcinidae	*Narcine entemedor*	1	1.2
Dasyatidae	*Neotrygon annotata*	1	1.2
Dasyatidae	*Neotrygon kuhlii*	1	1.2
Dasyatidae	*Neotrygon orientalis*	1	1.2
Odontaspididae	*Odontaspis ferox*	1	1.2
Orectolobidae	*Orectolobus hutchinsi*	1	1.2
Orectolobidae	*Orectolobus japonicus*	1	1.2
Orectolobidae	*Orectolobus ornatus*	1	1.2
Platyrhinidae	*Platyrhinoidis triseriata*	1	1.2
Pristiophoridae	*Pristiophorus cirratus*	1	1.2
Pristidae	*Pristis zijsron*	1	1.2
Somniosidae	*Proscymnodon plunketi*	1	1.2
Rhinobatidae	*Pseudobatos glaucostigma*	1	1.2
Rhinobatidae	*Pseudobatos lentiginosus*	1	1.2
Rhinobatidae	*Pseudobatos planiceps*	1	1.2
Rhinobatidae	*Pseudobatos prahli*	1	1.2
Rhinobatidae	*Pseudobatus productus*	1	1.2
Myliobatidae	*Pteromylaeus bovinus*	1	1.2
Rajidae	*Raja brachyura*	1	1.2
Rajidae	*Raja undulata*	1	1.2
Rhinobatidae	*Rhinobatos annulatus*	1	1.2
Rhinochimaeridae	*Rhinochimaera pacifica*	1	1.2
Myliobatidae	*Rhinoptera javanica*	1	1.2
Myliobatidae	*Rhinoptera steindachneri*	1	1.2
Carcharhinidae	*Rhizoprionodon porosus*	1	1.2
Rhinidae	*Rhynchobatus australiae*	1	1.2
Rhinidae	*Rhynchobatus djiddensis*	1	1.2
Rhinidae	*Rhynchobatus laevis*	1	1.2
Scyliorhinidae	*Scyliorhinus canicula*	1	1.2
Scyliorhinidae	*Scyliorhinus stellaris*	1	1.2
Sphyrnidae	*Sphyrna tiburo*	1	1.2
Rajidae	*Spiniraja whitleyi*	1	1.2
Squalidae	*Squalus acutipinna*	1	1.2
Squatinidae	*Squatina australis*	1	1.2

Stegostomatidae	*Stegostoma tigrinum*	1	1.2
Potamotrygonidae	*Styracura pacifica*	1	1.2
Dasyatidae	*Taeniura lessoni*	1	1.2
Triakidae	*Triakis semifasciata*	1	1.2
Urolophidae	*Trygonoptera ovalis*	1	1.2
Urolophidae	*Urolophus westraliensis*	1	1.2
Urotrygonidae	*Urotrygon aspidura*	1	1.2
Urotrygonidae	*Urotrygon chilensis*	1	1.2
Trygonorrhinidae	*Zapteryx xyster*	1	1.2
Rajidae	*Zearaja nasutus*	1	1.2
